# Attitudes and behaviors toward vaccination among nursing students from Spain and Portugal: a cross-sectional study

**DOI:** 10.1186/s12912-025-03627-3

**Published:** 2025-08-04

**Authors:** Francisco Javier Pérez-Rivas, Laura Esteban-Gonzalo, David García-García, María Julia Ajejas Bazán, Maria Clara Roquette-Viana, Adriana Dutra Tholl, Cristina Maria Alves Marques-Vieira

**Affiliations:** 1 Grupo de Investigación UCM “Salud Pública-Estilos de Vida, Metodología Enfermera y Cuidados en el Entorno Comunitario”, Departamento de Enfermería, Facultad de Enfermería, Fisioterapia y Podología, Universidad Complutense de Madrid, Madrid, 28040 Spain; 2 Instituto de Investigación Sanitaria Hospital 12 de Octubre (imas12), Madrid, 28041 Spain; 3 Grupo de Investigación Cuidados Avanzados de Enfermería, Department of Nursing and Nutrition, School of Biomedical and Health Sciences, Universidad Europea de Madrid, Madrid, 28670 Spain; 4 Academia Central de la Defensa, Escuela Militar de Sanidad, Ministerio de Defensa, Madrid, 28040 Spain; 5 Faculty of Health Sciences and Nursing, Centre for Interdisciplinary Health Research (CIIS), Universidade Católica Portuguesa, Lisboa, 1649-023 Portugal; 6 Nursing, Everyday Life, Imaginary, Health and Family Research and Innovation Laboratory of Santa Catarina (NUPEQUIS-FAM-SC), University of Santa Catarina, Florianópolis, 88035-97 Brazil; 7 Nursing School of Lisbon, Centre for Interdisciplinary Health Research (CIIS), Nursing Research, Innovation and Development Center of Lisbon (CIDNUR), Universidade de Lisboa, Lisboa, 1600-096 Portugal

**Keywords:** Vaccination, Vaccine hesitancy, Nursing students, University students, Attitudes, Behaviors, Health literacy, Spain, Portugal

## Abstract

**Background/Objectives:**

Since nursing students will be future promoters of immunization, it is essential to compare the attitudes and behaviors toward vaccination among nursing students in Portugal and Spain, to provide a comparative and contextualised view of the perceptions and practices of future health professionals in two countries with similar health systems and training structures, but influenced by different socio-cultural frameworks. Therefore, allows for the identification of similarities and divergences in the disposition towards vaccination, which is key for the design of more effective and culturally sensitive educational and public health strategies.

**Methods:**

This cross-sectional study assessed and compared attitudes and behaviors toward vaccination among nursing students from the Portuguese Catholic University (Lisbon, Portugal) and the Complutense University of Madrid (Spain). The study included 928 students from all four years of the nursing degree program, who completed the Questionnaire on Attitudes and Behaviors toward Vaccination in Health Sciences Students (ACVECS).

**Results:**

The results showed significant differences between both countries. Spanish students presented more favorable attitudes and behaviors toward vaccination compared to Portuguese students (*p* < 0.001). Women and native students obtained higher scores. Differences were observed according to academic year: in Spain, students in higher years showed better attitudes, while in Portugal it was first-year students who obtained higher scores. These differences could be influenced by cultural, educational, and pandemic-related factors.

**Conclusions:**

Spanish students showed significantly more favorable attitudes and behaviors toward vaccination than Portuguese students. Native students had a better attitude toward vaccination in both countries, this difference being especially notable in behaviors oriented toward vaccination among Portuguese students.

**Clinical trial number:**

Not applicable.

## Introduction

Vaccine hesitancy, understood as the refusal or delay in accepting vaccines despite the availability of vaccination services [1], represents a serious threat to the control of communicable diseases. Its impact on public health led the World Health Organization (WHO) to consider it one of the ten main threats to global health [2] and to integrate it as a priority challenge in the Immunization Agenda 2030: A global strategy to leave no one behind [3].

This phenomenon is influenced by multiple factors, including misinformation, lack of trust in the healthcare system, and personal beliefs [4]. In recent years, Larson and colleagues have pointed out the influence of other elements, such as growing distrust of experts, preference for alternative health approaches, political polarization, and belief-based extremism. Additionally, they highlight that since the COVID-19 pandemic, vaccine hesitancy has increased, largely due to the impact of media and the internet [5].

Healthcare professionals play a fundamental role in promoting immunization-related health literacy [6], as their ability to effectively communicate the benefits, risks, and safety of vaccines directly influences the confidence of parents and patients [5]. However, some professionals also exhibit vaccine hesitancy, which can range from doubts about the effectiveness of certain vaccines to openly anti-vaccination positions [7, 8].

Among healthcare professionals, nursing staff play a key role in combating vaccine hesitancy, as they are the group that dedicates the most time to direct patient care and education [9]. Their work is especially relevant in promoting vaccination, as they represent the main source of information and advice for the population [10]. Furthermore, their attitudes and behaviors regarding vaccines not only influence their own adherence to vaccination but also significantly impact their patients’ decisions [8].

## Background

Nursing professionals have a privileged position to influence several of the factors that determine vaccine hesitancy. Through effective communication, they can address knowledge gaps, modify negative attitudes, and strengthen commitment to vaccination. Their role is fundamental in optimizing vaccination services and addressing specific patient concerns [11].

The attitude of nursing students toward vaccination is a crucial factor, given that, as future healthcare professionals, they will play a key role in promoting immunization within public health programs [12, 13]. Understanding their attitudes toward vaccination is essential for identifying areas that require interventions, thus ensuring that students promote an informed and positive view of immunization in their future professional practice.

Recent international research confirms that attitudes toward vaccines among nursing students are diverse and influenced by a wide range of factors, including the academic year, prior clinical exposure, cultural context, perceived risk, and confidence in institutions. In a cross-sectional study conducted in Finland, Keisala et al. [14] identified four distinct vaccine attitude profiles among nurses and nursing students, highlighting the role of perceived vaccine safety and trust in health authorities. Among students, concerns regarding long-term side effects and limited trust in vaccine-related information were especially prevalent. Similar concerns about vaccine safety, effectiveness, and long-term effects have also been reported among nursing students in China [15], South Africa [16], and the United States [17], confirming that vaccine hesitancy is a recurrent issue across diverse cultural and educational contexts.

A particularly relevant and emerging concept is vaccine decision regret. Tayhan et al. [18], in a mixed-methods study with nursing and midwifery students, found that half of those vaccinated against COVID-19 reported regret, citing coercion, lack of information, and long-term safety concerns. Brera et al. [19] reinforced this finding among Italian students and nurses, where vaccine regret was associated with lower levels of trust and greater concern about vaccine safety. These experiences may influence future vaccination intentions and foster persistent hesitancy.

Sociodemographic and educational variables also shape students’ knowledge and acceptance. A study in Greece by Statiri et al. [20] showed low influenza vaccination uptake and modest knowledge levels among nursing students, with better results in those at more advanced stages of training. In Spain, Tuells et al. [21] found that while nursing students generally demonstrated favorable attitudes, their main information sources were informal, primarily family environments, and many expressed the need for more structured academic training on vaccines. Other studies highlight the impact of clinical exposure: students involved in COVID-19 care settings reported greater confidence in vaccination [22, 23].

In addition, several studies emphasize the importance of psychological, communicational, and contextual factors. Berry et al. [17] and Gabriel et al. [24] noted that misinformation, low perceived risk, and lack of institutional trust were significant predictors of hesitant behavior among students in Texas and Namibia. A global systematic review by Begum et al. [25] confirmed that vaccine acceptance is strongly shaped by cultural narratives, media influence, and the perceived credibility of information sources. Similarly, Choi and Ryu [26], in a meta-regression of 41 studies, found that vaccine acceptance varied significantly by geographic region and country income level, underscoring the importance of context-specific research to guide educational and public health strategies.

Although Spain and Portugal share EU membership and similar universal healthcare systems, their sociocultural contexts and vaccination-related educational approaches may differ. Previous studies have shown that vaccine confidence and risk perception vary even among European countries with comparable health infrastructures [27, 28]. This makes the comparison between these two countries particularly valuable for identifying educational and cultural determinants of hesitancy.

This research provides the first direct comparison of vaccination attitudes between nursing students in Spain and Portugal, two countries with similar healthcare systems but distinct sociocultural and political landscapes. By identifying country-specific determinants (e.g., political discourse, curricular differences), this study addresses gaps in understanding how regional contexts shape vaccine confidence among future health professionals. These findings enrich international literature by providing a framework to analyze cross-national variations in vaccine hesitancy and support the development of culturally sensitive strategies for health education and public health interventions in Europe.

Therefore, the objective of this study was to compare the attitudes and self-reported vaccination behaviors among nursing students in Spain and Portugal, and to explore the sociodemographic and academic factors associated with vaccine hesitancy in both countries. We hypothesized that, despite similarities in healthcare systems, there would be significant differences in vaccine confidence and behavior between students from both countries due to differences in cultural, educational, and political contexts.

## Methods

### Study design

A cross-sectional descriptive study was conducted to assess attitudes and behaviors toward vaccination among Portuguese and Spanish students enrolled in the Nursing Degree program, as well as to assess the differences between both populations. This study was prepared following the guidelines set out in the STROBE (Strengthening the Reporting of Observational Studies in Epidemiology) statement, to ensure transparency and quality in the presentation of results.

### Participants and recruitment

No sampling was carried out to select the sample. The entire available population was invited to participate, i.e. all students enrolled in the Nursing Faculties of both universities. Some visits were made by one of the researchers, who physically went to the classrooms, to encourage participation. Perhaps some of the students did not participate due to lack of time. The students who did not participate were not replaced, as from the beginning all the students enrolled were included and could not be replaced by others. They accessed the questionnaire through the University’s virtual campus. Those students who participated used a period of 10 to 15 min in the classroom using the Google Forms^®^ online platform. Students residing in Portugal were pursuing their nursing studies at the Portuguese Catholic University. For their recruitment, researchers contacted course coordinators and professors, collecting information after an awareness-raising activity in the classroom where the objectives of the study and the possibility of participating were presented. Subsequently, all information was sent by email where access to a link was attached that allowed them to respond to the online questionnaire, using Google Forms^®^. All information collected at both Universities was transferred to a common database, using Microsoft Office Excel^®^, 2016.

The inclusion criteria were (1) being enrolled in the Nursing Degree offered by the Faculty of Nursing, Physiotherapy, and Podiatry of the Complutense University of Madrid, or by the Lisbon School of Nursing, belonging to the Institute of Health Sciences of the Portuguese Catholic University, and (2) understanding Spanish/Portuguese to be able to complete the questionnaire. After carrying out the described procedure, all students who agreed to participate and gave their informed consent were recruited. Data collection took place between October 1 and November 22, 2021, for students enrolled at the Complutense University, and between March 25 and 31, 2022, for those enrolled at the Portuguese Catholic University. Complete data were obtained from a total of 928 students, 712 in Spain, out of 1024 students, and 216 in Portugal, out of 322 students. To estimate the necessary sample size, the formula for comparing means of two independent groups was used, assuming a common standard deviation of 0.46, based on a previous study [13]. Considering a significance level of 5%, a statistical power greater than 95%, and a ratio of 3 between the number of subjects in both groups (determined according to the number of students enrolled in each university), it was estimated that 651 subjects would be needed in the group of UCM students and 217 in the group of Portuguese students to detect a difference equal to or greater than 0.13 units in a two-tailed test. Consequently, the sample size achieved is adequate to meet the objectives of the study.

### Measurement instruments

#### Attitudes toward vaccination

Attitudes and behaviors toward vaccination were assessed using the ACVECS. This questionnaire was developed and validated by Fernandez-Prada et al. (2016) in university students [29]. It consists of a self-administered questionnaire with 24 items, with a Likert response scale from 0 to 4 (0 = strongly disagree, 1 = disagree, 2 = neither agree nor disagree, 3 = agree, 4 = strongly agree), which examines the beliefs, behaviors, and general attitude toward vaccination of students pursuing studies in the area of health sciences. The first 15 items examined the “beliefs” dimension, and the last nine examined the “attitudes” dimension. Together, these 24 items determined the “general attitude” toward vaccination. Items 1, 2, 7, 8, 15, and 23 were oriented in the opposite direction, so they were reversed for analysis. The total score was divided by the total number of items, forming a score between 0 and 4. A higher score is associated with more favorable attitudes toward vaccination. The scale has shown adequate levels of reliability and validity (Cronbach’s alpha 0.92). To avoid duplication or fraud in the online survey, the application did not allow only one survey to be sent per mail and IP address.

#### Biological and sociodemographic variables

Information was collected regarding age, sex, and country of origin. Age was reported by students in completed years. Sex was reported by inviting each student to choose the category of male or female. The country of origin was qualitatively reported by each student. Subsequently, a new variable was constructed with the categories of Spain/Portugal (native students), in case of having been born in those countries, and “other country” (non-native students). It is worth mentioning that students residing in Spain who indicated that their country of birth was Portugal, or vice versa, were included in the category “other country”.

##### Academic variables

Information was collected about the year/course of studies in which each student was enrolled. For this, each student was invited to choose among the categories of 1st-4th year.

### Ethical aspects

The protocol of this study was approved by the Research Ethics Committee of the San Carlos Clinical University Hospital of Madrid (Spain) (CI:20/376-E; May/2020) and by the Research Committee of the Portuguese Catholic University (CI:188; Feb/2022). All information regarding the objectives and procedure of the research was provided in writing to potential participants, in the form of an online form. Student participation was voluntary, with no compensation offered, and anonymity was guaranteed in the collection, sharing and use of the data. The data were managed exclusively by the principal investigator, who stored them in a password-protected database, ensuring the confidentiality and security of the information. Informed consent was obtained by express acceptance at the beginning of the questionnaire, where the objectives of the study were presented; only those participants who selected the ‘yes’ option in the first item were able to continue with the completion of the questionnaire, otherwise progress was impossible. Data were collected anonymously, respecting the confidentiality of the data collected in this study, in accordance with the Declaration of Helsinki.

### Data analysis

First, to describe the characteristics of the sample, as well as the results related to attitudes and behaviors toward vaccination, descriptive statistics were performed using mean and standard deviation for quantitative variables and absolute and relative frequency (percentages) for qualitative variables. Second, the distribution of variables was analyzed to evaluate their normal/non-normal distribution using the Kolmogorov-Smirnov test [30]. Third, a bivariate analysis was performed using the Mann-Whitney U and Kruskal-Wallis tests to assess associations between a quantitative variable and a qualitative variable with two or more categories, respectively, and Spearman’s correlation test to assess associations between quantitative variables (Table 1).

To analyse possible non-response bias, the socio-demographic and academic characteristics of the participants were compared with those of the total student population, and no statistically significant differences were observed. Likewise, early vs. late bias was assessed by comparing the initial responses with those received in later phases of data collection, without finding relevant variations in the main variables. Finally, the presence of common method bias was explored using confirmatory factor analysis and Harman’s one-factor test, finding that the variance explained by a single factor did not exceed the critical threshold of 50%, suggesting a low probability of this type of bias [31].

Third, a multilevel linear regression analysis with mixed effects was used to determine the associations between the scores of attitudes, behaviors regarding vaccination, as well as the total scores of the questionnaire used, and the study variable Portugal/Spain. Unadjusted and adjusted linear models were constructed by age, sex, and country of origin (Table 2).

This analysis strategy has been used with the aim of addressing the levels of grouping present among the participants examined, including in all models a random intercept for the variable year or course of studies (1st-4th) [32]. For each model, the Beta value with its standard error of each variable was calculated, with its confidence interval and the p-value of significance (*p* < 0.05) were considered statistically significant). The Statistical Package for the Social Sciences, version 25 (SPSS^®^. Inc., Chicago, USA) and STATA/SE 14.1 (Stata Corp LP) were used to develop the analyses described above.

## Results

The total response rate was 68.94%, very similar to the 69.53% obtained in Spain and 67.08% in Portugal.

The characteristics of the sample of Spanish students and the bivariate analysis are presented in Table 1. The scores for attitudes toward vaccination were higher among native students (born in Spain) (*p* < 0.001) and among students in more advanced years (3rd and 4th year) (*p* < 0.001). The score of the behaviors oriented toward vaccination dimension was higher in the group of women (*p* = 0.008). Finally, the general total score of the ACVECS questionnaire was higher in native students (born in Spain/Portugal) (*p* = 0.001), and in students from more advanced courses, 3rd and 4th year (*p* < 0.001).

**Table 1:** Characteristics of the participants examined

**Spanish sample**								
***n*** **=** **712**	***n***	%**/mean (SD)**	**Attitude score (mean, SD)**	***p*** ^**a**^	**Behavioral score (mean, SD)**	***p*** ^**b**^	**Total ACVECS score [mean, SD]**	***p*** ^**c**^
Total ACVECS score (0–4)	712						3.46 (0.38)	
Attitude score (0–4)			3.42 (0.38)					
Behavioral score (0–4)					3.51 (0.44)			
Age	712	21.8 (6.5)		0.281 (0.04)^x^		0.508 (-0.02)^x^		0.743(0.01)^x^
Sex				0.344^#^		**0.008** ^**#**^		0.060#
Women	607	85.3	3.43 (0.37)		3.54 (0.41)		3.48 (0.36)	
Men	105	14.7	3.35 (0.46)		3.37 (0.55)		3.36 (0.48)	
Country of origin				**< 0.001#**		0.204^#^		**0.001** ^**#**^
Spain	636	89.3	3.44 (0.39)		3.52 (0.43)		3.48 (0.38)	
Other	76	10.7	3.26 (0.33)		3.45 (0.48)		3.36 (0.38)	
Year of studies				< 0.001*		0.139*		< 0.001*
1º	171	24.0	3.33 (0.36)		3.47 (0.43)		3.40 (0.36)	
2º	150	21.1	3.38 (0.41)		3.47 (0.48)		3.43 (0.42)	
3º	194	27.2	3.50 (0.37)		3.55 (0.43)		3.52 (0.37)	
4º	197	27.7	3.44 (0.38)		3.54 (0.41)		3.49 (0.37)	
**Portuguese sample**								
***n*** **=** **216**	**n**	**%/mean (SD)**	**Attitude score (mean**,** SD)**	***p*** ^**a**^	**Behavioral score (mean**,** SD)**	***p*** ^**b**^	**Total ACVECS score [mean**,** SD]**	***p*** ^**c**^
Total ACVECS score (0–4)	216						3.27 (0.44)	
Attitude score (0–4)			3.21 (0.43)					
Behavioral score (0–4)					3.34 (0.52)			
Age	216	21.47 (4.64)		0.197 (-0.08)^x^		0.633(-0.03)^x^		0.321(-0.06)^x^
Sex				0.757#		0.961^**#**^		0.760^#^
Women	194	89.8	3.21 (0.43)		3.34 (0.51)		3.28 (0.44)	
Men	22	10.2	3.17 (0.46)		3.30 (0.62)		3.23 (0.50)	
Country of origin				**< 0.021#**		**0.016** ^**#**^		**0.016** ^**#**^
Portugal	198	91.7	3.23 (0.43)		3.37 (0.51)		3.30 (0.44)	
Other	18	8.3	2.97 (0.38)		3.04 (0.56)		3.01 (0.45)	
Year of studies				< 0.001*		0.706*		0.067*
1º	70	32.6	3.26 (0.43)		3.33 (0.54)		3.29 (0.45)	
2º	44	20.5	3.39 (0.37)		3.43 (0.43)		3.41 (0.38)	
3º	43	20.0	3.04 (0.44)		3.31 (0.50)		3.17 (0.44)	
4º	58	27.0	3.15 (0.41)		3.31 (0.56)		3.23 (0.46)	

P^a^ value for comparing age, sex and country of origin and Attitude score. P^b^ value for comparing age, sex and country of origin and Behavioral score. P^c^ value for comparing age, sex and country of origin and Total ACVECS score. ^X^ Spearman correlation test. # Mann–Whitney U-test. *Kruskal–Wallis test. ACVECS = Questionnaire on Attitudes and Behaviors Towards Vaccination in Health Sciences Students

The characteristics of the sample of Portuguese students and the bivariate analysis are presented in Table 1. The score of the attitudes toward vaccination dimension was higher in native students (born in Portugal) (*p* = 0.021) and in first- and second-year students (*p* < 0.001). The score of the behaviors oriented toward vaccination dimension was higher in native students (born in Portugal) (*p* = 0.016). Finally, the general total score of the ACVECS questionnaire was higher in native students (born in Portugal) (*p* = 0.016).

The mixed linear models for the associations between the ACVECS questionnaire score and the Spain/Portugal study variable are shown in Table 2. Given the absence of substantial differences between the adjusted and unadjusted model, this section will only describe the results associated with the model adjusted for age, sex, and country of origin. In this adjusted model, the score of the attitudes toward vaccination dimension was higher in Spanish students (b = 0.21 − 0.03 (SE), *p* < 0.001), compared to Portuguese students. Similarly, the score of the behaviors oriented toward vaccination dimension was higher in Spanish students (b = 0.17 − 0.03 (SE), *p* < 0.001). Accordingly, the general total score of the ACVECS questionnaire was higher in Spanish students (b = 0.19 − 0.03 (SE), *p* < 0.001), compared to the score obtained by their Portuguese counterparts.

**Table 2:** Linear regression mixes models for *Questionnaire on attitudes and behaviors towards vaccination in health sciences students (ACVECS)*

			Unadjusted model			Adjusted model^1^			
	*n*	Mean (SD)	β (SE)	95% CI	*P*	*n*	β (SE)	95% CI	*P*
*Attitude score*	927					927			
Spanish sample		3.42 (0.38)	0.20 (0.03)	0.14–0.26	**< 0.001**		0.21 (0.03)	0.14–0.27	**< 0.001**
Portuguese sample		3.21 (0.43)	-0.20 (0.03)	-0.26- -0.14	**< 0.001**		-0.21 (0.03)	-0.27- -0.14	**< 0.001**
*Behavioral score*	927					927			
Spanish sample		3.51 (0.44)	0.16 (0.03)	0.09–0.23	**< 0.001**		0.17 (0.03)	0.10–0.24	**< 0.001**
Portuguese sample		3.34 (0.52)	-0.16 (0.03)	-0.23- -0.09	**< 0.001**		-0.17 (0.03)	-0.24- -0.10	**< 0.001**
*Total ACVECS score*	927					927			
Spanish sample		3.46 (0.38)	0.18 (0.03)	0.12–0.24	**< 0.001**		0.19 (0.03)	0.13–0.25	**< 0.001**
Portuguese sample		3.27 (0.44)	-0.18 (0.03)	-0.24- -0.12	**< 0.001**			-0.25- -0.13	**< 0.001**

^1^ Adjusted Model: Adjusted for age, sex and country of origin

SD: standard deviation; SE: standard error

Note: Mean (SD) values are taken from Tables 1 and 2. Negative β coefficients for Portuguese sample indicate lower scores compared to Spanish sample (reference group)

## Discussion

This study has confirmed our hypothesis by revealing significant differences in attitudes and behaviors toward vaccination among nursing students from Spain and Portugal. Our findings support the notion that, despite similarities in healthcare systems and professional training, vaccine attitudes among future healthcare professionals are notably influenced by distinct socio‑cultural, educational, and political contexts.

Spanish nursing students exhibited significantly more favorable attitudes and behaviors toward vaccination than their Portuguese counterparts. These differences align with international evidence underscoring the role of context in shaping vaccine confidence. For instance, the State of Vaccine Confidence in the EU + UK report showed markedly higher trust among Spanish than Portuguese healthcare professionals [28], suggesting that the communication climate in Spain may be more conducive to fostering confidence in immunization [33]. Also, the more favourable socio-economic context in Spain may have contributed positively, as international studies have associated vaccine insecurity with lower economic status conditions [34].

Furthermore, in Portugal, the presence of political parties and public figures that during the COVID-19 pandemic adopted critical positions towards vaccination may have negatively influenced public perception and trust towards immunization campaigns. Parties such as Chega and National Democratic Alternative (ADN) disseminated messages that questioned the scientific evidence on COVID-19 and vaccines, sometimes even sharing misinformation through media and social networks [35]. In the Portuguese legislative elections held in 2025, these two parties together obtained 24.5% of the votes, reflecting a significant growth of political forces that have promoted critical or negationist discourses regarding the pandemic and vaccines. Previous studies have shown that political affiliation can predict intention to vaccinate, and that support for populist parties is associated with a lower intention to vaccinate and a higher proportion of people who believe that vaccines are neither safe nor necessary [36–38]. Regarding nationality, indigenous students scored higher on the dimension of attitudes towards vaccination in both Spain (*p* < 0.001) and Portugal (*p* = 0.021). For vaccination-oriented behaviours, the difference by nationality was significant only in Portugal (*p* = 0.016). These results are consistent with previous research that has documented a greater reluctance to vaccinate among people of foreign origin, often explained by socioeconomic factors, situations of vulnerability, language and cultural barriers, and lower trust in health systems [33, 39, 40].

The analysis by academic year showed divergent patterns. In Spain, third- and fourth-year students showed more favourable attitudes (*p* < 0.001), in line with studies linking clinical exposure and advanced education with greater vaccine literacy [14, 17, 20, 22, 39, 41], In contrast, in Portugal, it was first- and second-year students who exhibited more positive attitudes (*p* < 0.001). This pattern, where early-year students show greater vaccine acceptance, has also been observed in other contexts, such as China [15] and certain regions of Central Europe [42]. This difference may be related to academic fatigue, exposure to misinformation, or the experience of clinical placements during the health crisis [25, 26], as well as potential curricular differences between the two countries.

Regarding sex, Spanish women scored significantly higher on vaccination behaviours. The literature offers contradictory results: while some studies suggest that women are more likely to follow health recommendations because of a greater sense of responsibility towards health and family care [43, 44], others indicate that they are more reluctant due to concerns about possible adverse effects [45–47]. This complexity underlines the need for gender-sensitive strategies.

This study, however, has some limitations that should be considered when interpreting the results. The cross-sectional design precludes causal inference and the self-report nature of the questionnaire may introduce desirability bias. Contextual differences, such as the timing of data collection during the COVID-19 pandemic and differences in recruitment strategies between countries, may also have affected comparability. Additionally, no specific theoretical framework on vaccine acceptance or health behavior was incorporated, such as the WHO’s 3 C model [4] or the 5 C model of vaccine hesitancy [48], which limits the interpretation of findings within broader explanatory models. Finally, relevant contextual variables such as socioeconomic status, religious beliefs, or detailed information on curricular vaccination content were not included, limiting the explanatory depth of the analysis.

### Implications for nursing education and practice

The findings point to several concrete, evidence‑informed actions that can be incorporated into undergraduate nursing curricula and continuing professional development:

1. Structured vaccinology modules that anticipate emerging threats. Core content should go beyond routine schedules to include principles of pandemic preparedness, risk communication, cold‑chain logistics and infodemic management, drawing on lessons learned from COVID‑19 and recent zoonotic outbreaks [3, 11].
2. Active learning strategies that mirror real‑world vaccination scenarios. Highfidelity simulation of massvaccination clinics, teambased learning around outbreak case studies, and flippedclassroom debates on vaccine misinformation have been shown to improve knowledge, confidence and uptake intentions [49]. Virtual‑ or augmented‑reality tools allow students to rehearse intramuscular injection technique, cold‑chain breaches and triage during supply shortages without compromising patient safety [50].
3. Peer‑led and service‑learning approaches to foster collective responsibility. Role‑playing, peer teaching and supervised participation in community vaccination drives encourage reflexive dialogue and strengthen pro‑social attitudes toward immunization [51].
4. One‑Health and climate‑sensitive perspectives. Integrating content on zoonotic spill‑over, vector‑shift due to climate change and the nurse’s role in cross‑sectoral surveillance prepares graduates for future natural hazards and pandemics [52].
5. Culturally tailored counselling and multilingual resources. Given the lower scores among students of foreign origin, curricula should include communication scripts adapted to diverse belief systems and language needs, as well as opportunities for intercultural exchange during clinical placements.

These targeted, interactive methods can strengthen vaccination competencies from the first year of training and build a workforce capable of responding rapidly and confidently to future public‑health emergencies.

## Conclusions

This study demonstrates that nursing students’ attitudes and behaviours toward vaccination are shaped by a complex interplay of socio‑cultural, educational and political factors, even across neighbouring countries with comparable health‑care systems. Country of training and student origin consistently influenced attitudes and behaviours, while academic progression and sex showed more context‑dependent patterns. These insights reinforce the need to embed contextual variables into vaccinology teaching and professional‑development strategies.

To translate these findings into practice, nursing programmes should prioritise the development of structured vaccinology modules. These should be complemented by active learning methodologies, such as peer learning, service-learning and scenario-based simulation, that can contribute to improving students’ attitudes and behaviours toward vaccination. Once these foundational competencies are established, broader content related to pandemic preparedness, zoonotic spill-over and climate-sensitive health risks may be progressively incorporated to strengthen public-health responsiveness. Particular attention should be paid to culturally tailored counselling resources for students of foreign origin.

Future studies should employ longitudinal and intervention designs to test the effectiveness of these educational approaches and to explore how broader political and social climates modulate vaccine confidence over time. The evidence generated by such studies can serve as a foundation for developing policies that better prepare nursing professionals for both routine immunisation and rapid responses to future public health emergencies.

## Data Availability

The data presented in this study are available upon request from the corresponding author.

## Abbreviations

ACVECS: Questionnaire on Attitudes and Behaviors toward Vaccination in Health Sciences Students
SE: Standard Error
SD: Standard Deviation
WHO: World Health Organization
